# Frequency of Cardiovascular Manifestation in Patients With Rheumatoid Arthritis

**DOI:** 10.7759/cureus.14631

**Published:** 2021-04-22

**Authors:** Pardeep Kumar, FNU Kalpana, Manoj Kumar Khamuani, Sameer Lohana, Suman Dembra, Maha Jahangir, Faryal Anees, Besham Kumar

**Affiliations:** 1 Medicine, Liaquat University of Medical and Health Sciences, Jamshoro, PAK; 2 Internal Medicine, Shaheed Mohtarma Benazir Bhutto Medical University, Larkana, PAK; 3 Medicine, Peoples University of Medical and Health Sciences for Women, Nawabshah, PAK; 4 Anesthesiology, Dow University of Health Sciences, Karachi, PAK; 5 Obstetrics and Gynecology, Agha Khan University Hospital, Karachi, PAK; 6 Internal Medicine, Jinnah Sindh Medical University, Karachi, PAK; 7 Internal Medicine, Jinnah Postgraduate Medical Centre, Karachi, PAK

**Keywords:** extraarticular manifestation, rheumatoid arthritis, patient characteristics

## Abstract

Introduction

Rheumatoid arthritis (RA) is a chronic, inflammatory, systemic autoimmune disease. The increased inflammatory burden in RA may result in atherosclerosis, myocardial infarction (MI), and subsequent mortality. In this study, we will determine the frequency of cardiovascular manifestation in RA patients through history, laboratory workup, and echocardiography.

Methods

This cross-sectional study was conducted in the rheumatology unit of a tertiary care hospital in Pakistan. Three hundred and twenty-two (n=322) participants with a previously confirmed diagnosis of RA were enrolled in this study via consecutive convenient non-probability sampling.

Results

Cardiovascular manifestations were present in 188 (58.3%) participants. More participants had positive rheumatoid factor (82.9% vs. 32.8%; p-value: < 0.0001) in RA patients with cardiovascular manifestation compared to RA patients without cardiovascular manifestation. Patients with cardiovascular manifestations have a significantly higher C-reactive protein (CRP; 10.21 ± 2.81 mg/L vs. 8.17± 2.01 mg/L; p value: < 0.0001) and erythrocyte sedimentation rate (ESR; 16.2 ± 3.14 mg/L vs. 15.1 ± 2.99 mg/L; p value: 0.0017).

Conclusion

In this study, patients with a cardiovascular manifestation had a higher frequency of patients with rheumatoid factor, higher mean values of CRP and ESR. The early diagnosis and management of cardiac manifestations would aid in controlling the severity of the disease and the overall mortality.

## Introduction

Rheumatoid arthritis (RA) is a chronic, inflammatory, systemic autoimmune disease. It occurs more frequently in females as compared to males, predominantly targeting the elderly [1]. Symptoms of RA include morning stiffness of the affected joints for >30 minutes, tender swollen joints, fever, weight loss, and rheumatoid nodules under the skin. However, the diagnostic criteria is undifferentiated arthritis involving three or more joints, positive rheumatoid factor (RF), and/or anti-citrullinated peptide/protein antibody, disease duration of more than six weeks, and elevated C-reactive protein (CRP) or erythrocyte sedimentation rate (ESR) [2-3]. RA primarily affects the lining of the small synovial joints, progressing to larger joints, eventually involving different body organs and causing progressive disability [1].

Various studies in the past have been conducted to determine the systemic manifestations of RA, among which cardiovascular diseases (CVD) have been found to contribute to the highest rate of mortality and morbidity [4]. The increased inflammatory burden in RA causes early atherosclerosis, stiffening, and calcification in the arterial system, leading to myocardial infarction (MI) and subsequent mortality [5-6]. However, there is high uncertainty that clinical cardiac manifestations are independent or concurrent with the RA manifestations [5]. Since many of these manifestations remain clinically silent for a longer period, modifiable risk factors should be identified early and treated. These risk factors include smoking, obesity, increased serum total cholesterol and low-density lipoprotein, diabetes mellitus, hypertension (HTN), and physical inactivity. Emerging risk factors include low serum high-density lipoprotein, increased serum triglycerides, CRP, and seropositivity to RF [6-7].

In this study, we aim to determine the frequency of cardiovascular manifestation in RA patients through history, laboratory workup, and echocardiography. Early recognition and management of traditional cardiovascular risk factors is essential to identify cardiovascular manifestations in these patients.

## Materials and methods

This comparative cross-sectional study was conducted in the rheumatology unit of a tertiary care hospital in Pakistan from June 2019 to May 2020. Three hundred and twenty-two (n= 322) participants with a previously confirmed diagnosis of RA were enrolled in this study via consecutive convenient non-probability sampling. Patients with hypertension and a previous history of myocardial infarction were excluded from our study. Ethical review board approval was taken before the enrolment of patients.

After informed consent, the participant’s age, time since diagnosis, gender, and rheumatoid factor status was noted. Phlebotomy was done to draw blood and send it to the laboratory for CRP or ESR. With the help of cardiologists, cardiovascular auscultation was done to identify any abnormal heart sound. Chest X-ray, electrocardiogram (ECG), and echocardiography were done to identify various abnormalities. Chest X-ray was used to look for any effusions. Echo was used to detect any valvular disorder, motion abnormalities, wall thinning, and cavity dilatation. ECG was used to look for abnormalities, including abnormalities in the QRS complex and ST segment and changes in the P and T waves. Participants with cardiovascular manifestations were identified as the case group.

Cardiovascular manifestations included pericarditis, cardiomyopathy, valvular heart diseases, and ECG abnormalities. A diagnosis of pericarditis was made with findings from ECG, echocardiography, and chest X-ray. Chest X-ray and echocardiography were used to detect fluid in the pericardial sac. ECG findings of pericarditis included upward ST-segment elevation and PR-segment depression in leads II and V3. Echocardiography was used to identify cardiomyopathy by looking at ventricular dilation, systolic and diastolic dysfunction, wall thickness, and impaired contractility. Echocardiography was also used to identify valvular heart disease by looking at the narrowing or calcification of valves, flow of blood, and restricted motion of valves. ECG abnormalities included isolated changes, without correlating findings in echo and chest X-ray, such as a bifid P wave, inverted T or P wave, narrow or broad QRS complex, prolonged or shortened PR interval, and ST-segment changes. ECG abnormalities are furthered defined in Table 1.

**Table 1:** Definitions of ECG abnormalities Abbreviation: ECG, electrocardiogram; mV, milliVolt; mm, millimeter

ECG abnormalities	How they were defined in this study
ST-segment Elevation	Horizontal or upsloping ST elevation ≥ 1.0 mm
ST-segment Depression	Horizontal or downsloping ST depression ≥ 0.5 mm
QRS-segment broad	QRS duration of greater than 0.12 seconds (more than 3 small boxes)
QRS Segment narrow	QRS duration of less than 0.08 seconds (less than 2 small boxes)
PR interval prolonged	PR interval more than 0.2 seconds (more than 5 small boxes)
Short PR interval	PR interval less than 0.12 seconds (less than 3 small boxes)
Peaked P wave	Longer than 0.08 seconds (More than 2 small boxes)
Abnormal P wave	Negative terminal component of the P wave exceeds 0.04 seconds in duration (equivalent to one small box)
Inverted T waves	Inversion of T wave is deeper than 1.0 mm
Peaked T waves	T waves look like isosceles triangles
Low voltage T waves	T waves less than 1mV in the limb leads and less than 2mV in the precordial leads

The Statistical Package for the Social Sciences® software version 23.0 (IBM Corp., Armonk, NY) was used for data analysis. For numerical variables, data such as age, CRP, and ESR were expressed as mean ± standard deviation. Frequencies and percentages were used for categorical variables such as gender and symptoms. The independent t-test and chi-square test were applied to compare participants with cardiovascular manifestation and participants without cardiovascular manifestation. A p-value of less than 0.05 meant that there is a difference between the two groups and the null hypothesis was not valid.

## Results

Cardiovascular manifestations were present in 188 (58.3%) participants. The mean age of participants and the year since the diagnosis of disease were comparable between groups with and without cardiovascular manifestations. More participants were RF positive (82.9% vs. 32.8%; p-value: < 0.0001) in RA patients with cardiovascular findings compared to RA patients without cardiovascular manifestations. Patients with cardiovascular manifestations had a significantly higher C-reactive protein (10.21 ± 2.81 mg/L vs. 8.17± 2.01 mg/L; p-value: < 0.0001) and erythrocyte sedimentation rate (16.2 ± 3.14 mg/L vs. 15.1 ± 2.99 mg/L; p-value: 0.0017) (Table 2).

**Table 2:** Comparison between rheumatoid arthritis patients with and without cardiovascular manifestations RA, rheumatoid arthritis; RF, rheumatoid factor; CRP, C-reactive protein; ESR: erythrocyte sedimentation rate

Characteristics	RA Patient With Cardiovascular Manifestations (n=188)	RA Patient Without Cardiovascular Manifestations (n=134)	p-value
Age in years (mean ± standard deviation)	41 ± 12	43 ± 13	0.15
Year since diagnosis (mean ± standard deviation)	4.5 ± 1.2	4.2 ± 2.1	0.1
Female (%)	121 (64.3)	79 (58.9)	0.32
RF Positive (%)	156 (82.9)	44 (32.8)	< 0.0001
CRP (mg/L)	10.12 ± 2.81	8.17± 2.01	< 0.0001
ESR (mg/L)	16.2 ± 3.14	15.1 ± 2.99	0.0017

The most common cardiovascular findings were ECG abnormalities in 102 (54.2%) participants, followed by pericarditis (48.9%). Valvular heart diseases (VHDs) were found in 92 (48.9%) participants, the most common being mitral regurgitation (Table 3).

**Table 3:** Spectrum of cardiovascular manifestations in patients with rheumatoid arthritis AR, aortic regurgitation; AS, aortic stenosis; ECG, electrocardiogram; MR, mitral regurgitation; VHDs, valvular heart diseases

Cardiovascular Manifestations	Frequency (%)
Pericarditis	92 (48.9)
Cardiomyopathy	61 (32.4)
ECG abnormalities	102 (54.2)
VHDs	92 (48.9)
MR	51 (55.4)
AR	38 (41.3)
AS	03 (3.2)

## Discussion

Rheumatoid arthritis, a chronic inflammatory disease, has an estimated annual incidence of 0.3%-1.0 % [8]. It is majorly related to the joints, but it also affects other systems of the body causing extra-articular manifestations (EAMs) like rheumatoid nodules, pulmonary involvement, or inflammation of the blood vessels [9]. EAMs in RA have been frequently observed in approximately 18-41% of the patients [10]. The most common of these manifestations are the rheumatoid nodules [11] that have been seen in 7% of RA patients when it is diagnosed [12], and approximately 30% occur during the disease development [13]. RA patients with rheumatoid nodules and long-established disease are more likely to report severe EAMs like vasculitis, rheumatoid lung disease, pericarditis, and pleuritis [14], and patients who develop rheumatoid nodules during the first two years after the diagnosis of RA are more prone to get severe EAMs [15]. However, in some patients, severe EAMs, such as interstitial lung disease and serositis, are observed even before the onset of joint symptoms [16].

In our study, RF positivity was observed more in RA patients with cardiovascular manifestations (82.9%) as compared to those without these manifestations (32.8%). Elevated levels of CRP and ESR were found in patients with cardiovascular manifestations. Among the cardiovascular manifestations, ECG abnormalities were the most commonly reported, followed by pericarditis and VHDs. Among VHDs, mitral regurgitation was found to be the most common, followed by aortic regurgitation. It is believed that abnormal echoes from the valve in RA patients indicate that the valves have undergone fibrosis due to extra-articular inflammation [17]. Choy et al. stated that pericarditis is one of the leading heart-related complications in RA patients and damages to the heart valves are frequently observed in patients with RA [18].

CVDs are known to be one of the most common causes of death in RA patients, with a 50% higher risk than the normal population [19]. Researchers are of the opinion that RA and CVD are common in pathogenesis in terms of their mechanisms of inflammation and immunity [19]. RA is known to speed up atherosclerosis; this idea has been anchored by research conducted on RA patients at three years of follow-up. The study showed that systemic inflammation causes thickening of carotid intima-media that is a potential marker of subclinical atherosclerosis [19]. However, more research is needed to explore the exact cause of increased cardiovascular risk in RA patients [20].

To the best of our knowledge, this is the first study that studies the cardiovascular manifestation of rheumatoid arthritis in the local population. The limitations of our study include that since it was a cross-sectional study, associations between RA and cardiovascular manifestation could not be established. It was a single-center study, making the diversity of the sample limited. Based on finding our study, it is highly recommended that cardiologists should be consulted during the management of rheumatoid arthritis. This may assist in the early identification and appropriate management of cardiovascular symptoms associated with rheumatoid arthritis, reducing the disease burden in patients with RA.

## Conclusions

In this study, patients with cardiovascular manifestations had a higher frequency of patients with rheumatoid factor and higher mean values of CRP and ESR. Our study primarily focused on the high prevalence of cardiac manifestations in RA patients and higher values of parameters, which are associated with poor prognosis in patients with cardiovascular manifestations. The early management of cardiac manifestations would aid in controlling the severity of the disease and overall mortality.
